# Can Social Functioning in Schizophrenia Be Improved through Targeted Social Cognitive Intervention?

**DOI:** 10.1155/2012/742106

**Published:** 2012-06-13

**Authors:** David L. Roberts, Dawn I. Velligan

**Affiliations:** Division of Schizophrenia and Related Disorders, Department of Psychiatry, University of Texas Health Science Center, 7703 Floyd Curl Drive, MC 7797, San Antonio, TX 78229, USA

## Abstract

Efforts to use cognitive remediation in psychosocial intervention for schizophrenia have increasingly incorporated social cognition as a treatment target. A distinction can be made in this work between “broad-based” interventions, which integrate social cognitive training within a multicomponent suite of intervention techniques and “targeted” interventions; which aim to enhance social cognition alone. Targeted interventions have the potential advantage of being more efficient than broad-based interventions; however, they also face difficult challenges. In particular, targeted interventions may be less likely to achieve maintenance and generalization of gains made in treatment. A novel potential solution to this problem is described which draws on the social psychological literature on social cognition.

## 1. Introduction

Over the past twenty years, it has become clear that front line treatments for schizophrenia, in particular medication, do not yield sufficient improvement in functional outcome in this population [1]. Thus, treatment developers have sought new intervention approaches. Prominent among these has been neurocognitive training (We use the term “neurocognitive” rather than “cognitive” to draw a clearer contrast for the reader between neurocognition and social cognition.), which aims to improve basic cognitive functions (e.g., attention, memory, and executive function) through compensatory strategies and/or remediative practice. Although still a relatively young field, research now suggests that neurocognitive training can enhance cognitive functioning among individuals with schizophrenia, and there is growing evidence that it can improve functional outcomes [2, 3]. Importantly, the effect of neurocognitive training on functional outcomes appears to be greatest when it is bundled within a broader treatment package that includes more functionally proximal interventions, such as vocational placement [4].

Recognizing the importance of targeting functionally proximal domains in treatment, some researchers have incorporated *social cognition* as an intervention target in psychosocial treatment for schizophrenia. Social cognition refers to the mental operations underlying interpersonal functioning [5]. In schizophrenia research, it most often is seen as comprising *emotion perception* (the ability to infer others' emotional states), *theory of mind* (ToM; the ability to infer others' mental states), and *attributional bias* (individual tendencies in explaining the causes of social events [6]). As a treatment target, social cognition has the advantage of being conceptually more proximal to, and more strongly correlated with, social functioning than are traditional neurocognitive domains [7]. Social cognition also appears to mediate the relationship between neurocognition and social functioning [8]. Thus, social cognition is a highly promising treatment target for improving social functioning in schizophrenia.

Despite the promise of social cognitive intervention, this is a young research area that is facing several important obstacles. These include inconsistency in the conceptualization and measurement of social cognition [9] and equivocal support for the efficacy of emerging social cognitive interventions [10, 11]. The current paper addresses one particular problem facing social cognitive intervention research: the generalization and maintenance of treatment gains. Specifically, we examine whether improvements in social cognition generalize to social functioning improvements and are maintained through time, or whether it may be necessary to bundle social cognitive intervention with behavioral interventions, as has been successful in neurocognitive training programs.

## 2. Social Cognitive Intervention for Schizophrenia

To date, social cognitive intervention techniques have combined elements of neurocognitive training with elements of cognitive psychotherapies. Elements adapted from neurocognitive training include highly domain-specific computerized drill-and-repeat practice, as in various face emotion perception training programs (e.g., [12]). Cognitive therapy elements include graded confidence judgments (e.g., “I am 70% sure that the woman in the picture is happy.”) and psychoeducation regarding the interaction of thoughts, cognitions, and feelings (e.g., [13]). However, social cognitive interventions can be distinguished from traditional cognitive therapy in the former's emphasis on cognitive process rather than content. Where cognitive therapy places relatively greater emphasis on static beliefs (e.g., the core belief, “I am unlovable.”), social cognitive therapy places greater emphasis on content-neutral processing capacities (e.g., the ability to infer mental and emotional states) and processing biases (e.g., the tendency to jump to conclusions [14]).

Social cognitive interventions can be divided roughly into *broad-based* and *targeted* interventions [15]. Broad-based interventions combine social cognitive treatment with social skills training, neurocognitive training, case management, and other intervention techniques. As such, they overlap considerably with the type of successful broad-based neurocognitive training packages noted above and have the potential to enjoy the same benefits to generalization and maintenance of gains that are conferred through such intensive and multilevel intervention packages. On the other hand, targeted social cognitive interventions focus treatment solely on the remediation of one or more social cognitive domains at the exclusion of neurocognition, behavioral social skills training, or other intervention modalities. There is hope that these targeted approaches may confer greater benefit to social functioning than targeted neurocognitive approaches due to the closer relationship of social cognition to social functioning [6].

## 3. Broad-Based Interventions

The first modern interventions to attempt to remediate social cognition in schizophrenia were broad-based interventions, including Cognitive Enhancement Therapy (CET [16]) and Integrated Psychological Therapy (IPT [17]).

CET and IPT differ from one another in several respects, such as the fact that CET is built on a neurodevelopmental model while IPT's theoretical model is not developmental. However, the common features of CET and IPT are more salient in summarizing their role in the broader literature. Both espouse a hierarchical model in which neurocognitive abilities are seen as a foundation upon which high-level social cognitive skills are built. In IPT, social cognition is explicitly posited as mediating the relationship between neurocognition and functional outcome [18], a model that has received growing empirical support in the research literature [8]. In both CET and IPT, neurocognitive intervention takes place largely through computer-based training while social cognitive intervention is provided via group didactics and exercises designed to capitalize on secondary socialization and group process. Both conceive of social cognitive improvement as emerging from the combination of highly specific training exercises and mechanistically diffuse social learning experiences. Through this combination, it is hoped that patients will build specific capacities while also gaining the flexibility and “wisdom” necessary to navigate an ever-changing social world in which the rigid lessons of primary socialization (e.g., *always* say “please” and “thank you”) are not sufficient [19].

Several intervention techniques from CET and IPT have been widely adapted in subsequent social cognitive interventions and are illustrative of typical intervention approaches in the field. For example, IPT uses a stepwise progression from conceptually simpler and less emotionally evocative training elements (e.g., guessing the intention of a character in a comic strip) to more complex and potentially emotionally arousing elements (e.g., learning to regulate one's own emotions). In CET, the final phase of social cognitive treatment is designed to help participants to generalize social cognitive skills to their everyday life. This approach is used in subsequent targeted interventions such as Social Cognition and Interaction Training (SCIT [13]), in which participants are taught to apply newly acquired social cognitive skills to upsetting or confusing social events in their day-to-day lives. IPT also uses a range of social stimuli—including photographs, audio and written vignettes, and videos; this use of social stimuli has become the standard for later social cognitive interventions (e.g., [13, 20–23]).

Another technique originally developed in IPT that has subsequently been adapted in newer social cognitive interventions (e.g., [13, 23]) is the practice of distinguishing between the facts of a social situation and one's interpretation of those facts. For example, a photograph of a person receiving a gift may include the *fact* of the recipient smiling, which may lead to the participant's *interpreting* the recipient as feeling happy. Distinguishing between facts and interpretations is widely seen as a core skill that spans the link between social cognition and metacognition. It requires metacognitive awareness to recognize that a strongly held view may be an interpretation rather than a fact, as well as metacognitive control to inhibit endorsing an interpretation as if it were a fact [14].

Separating facts from interpretations is at the core of a broader family of intervention techniques that can be termed the “social detective” approach [15] which is used in a range of targeted interventions. This approach teaches patients to analyze social situations carefully and systematically, as if they were detectives. In addition to separating facts from interpretations, patients are taught to spend time gathering additional behavioral and contextual information to use as a basis for judging their confidence in competing explanatory hypotheses. The social detective approach is applied to multiple social cognitive domains. In emotion perception training, patients are taught to interpret facial expressions (e.g., smile, raised eye brows) as “clues” as to the underlying emotion that a person may be feeling [13]. In ToM training, patients are taught to use contextual facts (e.g., a birthday cake on the table) as a basis for predicting people's thoughts and feelings (surprise, happiness). And in attributional bias and jumping-to-conclusions training, patients are taught that a careful, logical approach can buffer them against the perils of hasty judgment [14].

Both CET and IPT have shown moderate to strong evidence of improving both social cognition and social functioning [24, 25]. The effects of CET and IPT on social functioning likely result from the combination of social cognitive training with other treatment elements, including social skills training and neurocognitive training. Mechanistically, this may work in several ways. For example, improvements to memory may enable patients to retain learned social cognitive skills for longer and to recall and deploy these skills when necessary in real-world contexts. Social skills training provides opportunities to practice drawing on improved memory and social cognitive skills in vivo.

By addressing social cognition as one of several treatment targets, CET and IPT leave unanswered a central question of the social cognitive treatment project: is it possible to improve social functioning solely by way of improving social cognition? That is, can the strong and independent relationship between social cognition and social functioning be leveraged such that interventions which target social cognition at the exclusion of neurocognition lead to enhanced functioning? This question is important practically because interventions such as CET and IPT are labor, time, and resource intensive, rendering them unfeasible in a range of treatment settings. In contrast, targeted social cognitive interventions tend to be much simpler, less expensive, and thus potentially available to a wider cross-section of patients [26].

## 4. Targeted Interventions

The first interventions to target social cognition at the exclusion of other domains were laboratory-based proof-of-concept trials that demonstrated the modifiability of social cognition through highly specific interventions but did not evaluate the long-term maintenance of improvement or the effect of improved social cognition on social functioning (e.g., [12, 27–30]). The majority of these interventions targeted the social cognitive domain of face emotion perception, which lends itself to drill-and-repeat practice. For example, Silver et al. [27] demonstrated that it is possible to improve schizophrenia patients' performance on tests of emotion perception through repeated practice judging still images of human faces. However, the authors did not evaluate the generalization of these improvements to social functioning. In a refined approach, Wölwer et al. integrated this type of drill-and-repeat practice with principles of errorless learning, verbalized self-instruction, feature abstraction, and positive reinforcement [12]. In a treatment trial comparing this intervention to neurocognitive training, emotion perception training led to significant improvements in emotion perception but not in neurocognitive domains. Meanwhile neurocognitive training led to improvements in verbal memory but not emotion perception [28]. As in the Silver study, generalization to functional outcome was not assessed.

Beginning in the early 2000's, targeted social cognitive interventions expanded beyond narrow laboratory trials, intervening on a wider spectrum of social cognitive domains and endeavoring to link social cognitive gains to improved social functioning. For example, SCIT and Social Cognitive Skills Training (SCST [23]) target emotion perception, as well as ToM, jumping to conclusions and attributional style and also include intervention techniques designed to aid patients in applying improved social cognition to real-world social functioning.

SCIT is designed as a weekly group intervention lasting for approximately 20–24 sessions. It is divided into three phases which, like IPT, are designed to be easier and less stressful early on and to become increasingly difficult and emotionally challenging later. Phase I of SCIT introduces the concept of social cognition and provides emotion perception training that draws heavily on techniques validated in laboratory settings. These include drill-and-repeat practice, as well as psychoeducation, facial mimicry [31], and attention shaping toward emotion cues [32]. Phase II of SCIT addresses ToM deficits and attributional bias using a combination of established techniques, such as separating facts from interpretations, and novel techniques. For example, patients play a modified form of the game *20 Questions *to improve their data gathering within a social detective framework. Departing from laboratory-based-targeted interventions, Phase III of SCIT provides patients with a set of techniques for applying social cognitive skills in their day-to-day lives. During this phase, patients bring examples of real interpersonal difficulties from their lives into the SCIT group and use an integrated technique to evaluate the situation, make an action plan to improve understanding and reduce interpersonal distress, and practice the action plan during group.

SCST consists of 12 weekly group sessions that integrate didactic presentation and exercises. Sessions are divided into a first phase, addressing emotion and social perception and a second phase, addressing social attribution and Theory of Mind. Like SCIT, SCST incorporates established training techniques from previous targeted interventions (e.g., [13, 16]). SCST also expands upon previous interventions in several ways, including newly developed training exercises to enhance recognition of behavioral social cues, such as gesture, and a range of new pictorial, video, and audio stimuli.

SCIT, SCST, and other similar interventions have shown evidence of efficacy in improving both social cognition and social functioning (e.g., [33]); however, results have not been uniformaly positive (e.g., [23, 34]). Given the nascent status of the field of targeted social cognitive intervention, it is a high priority to address challenges facing these early targeted interventions in order to refine and improve them [35].

## 5. Challenges Facing Targeted Interventions

In order for a psychosocial intervention to meaningfully improve a patient's social functioning, gains must generalize to the range of social and community situations encountered by the patient, and gains must also be maintained through time. Social cognition is a promising treatment target from the standpoint of generalization, because the domain is conceptually proximal to and statistically correlated with global measures of social functioning [7]. Thus, theoretically, social cognitive gains may confer real-world benefits across social domains with limited need for further generalization tools.

However, training will only yield real-world functional improvement if patients are able to maintain and apply social cognitive gains in real-world contexts. Due to cognitive deficits, individuals with schizophrenia often struggle to transport skills and lessons from the treatment environment to their day-to-day lives [36]. This hurdle has been addressed in at least three ways in existing psychosocial interventions: (1) Neurocognitive training, (2) overlearning, and (3) environmental supports.

*Neurocognitive training* may improve patients' ability to transport and apply lessons via improved cognitive abilities [37]. Thus, enhanced memory function may improve patients' ability to recall strategies and to deploy them across varied real-world situations. Similarly, enhanced executive functions may improve patients' ability to use skills flexibly, enabling them to apply skills across a range of real-world settings. These benefits of neurocognitive capacity may contribute to the beneficial effects of neurocognitive training approaches, especially when combined with other interventions, including broad-based social cognitive treatments like Cognitive Adaptation Training (CAT [38]) and IPT. Unfortunately, targeted social cognitive interventions do not aim to improve neurocognition, and there is little evidence that they yield improvement in this domain ([12]; although see [33]).

 Regarding *overlearning*, Kern et al. [39] have suggested that drill-and-repeat approaches lead to maintenance of skill by shifting demand from explicit, controlled processing, which is typically deficient in schizophrenia, to implicit, automatic processing, which is relatively intact [40]. Through repetition, using overlearning and errorless learning principles, patients have been able to learn vocational and interpersonal tasks to the point of relative automaticity and to maintain these gains through time [39, 41]. Overlearning techniques have been used in targeted social cognitive interventions, primarily in emotion perception training. There is evidence linking these interventions to skill maintenance (e.g., [35]); however, to date, there is not compelling evidence that improving the highly circumscribed skill of face emotion perception leads to improved social functioning [26].

Drill-and-repeat techniques have been used in interventions that target ToM [42] as well as interventions that target multiple social cognitive domains (e.g., [13, 23]). However, these applications have struggled to operationalize the skill being taught with sufficient specificity, or to include sufficient repetition, to achieve true automatization of the targeted skill. For example, participants in SCIT learn the skill of separating facts from interpretations (described above) in order to decrease attributional bias and jumping to conclusions. However, this skill is taught more as a psychoeducational lesson about the importance of not jumping to conclusions, with practice conducted to ensure comprehension rather than to achieve automaticity of the skill.

It is unlikely that this limitation could be fixed simply by increasing repetition of ToM and attributional bias interventions because these interventions are conceptually incompatible with the goal of achieving automaticity of skill. These interventions teach patients to slow down their thinking and emphasize conscious, careful deliberation in order to avoid mistakes and maximize accuracy (e.g., [14, 42]). By definition, slow, controlled thought cannot be done quickly and automatically [43].

*Environmental supports* are a third approach to facilitate the maintenance of treatment gains in patients' community living environments. Whereas neurocognitive training and overlearning improve patients' ability to recall and deploy acquired skills, environmental interventions, such as Cognitive Adaptation Training [38], modify patients' physical environments in order to bypass cognitive deficits and cue adaptive functional behavior. For example, a specialized alarm may sound the verbal alert, “It is 10 AM, remember to take your medication.” For patients with diminished inhibitory control, their home environment may be decluttered to decrease distractors. For patients with prominent apathy, important functional items, such as clothing and hygiene products, may be made more visible and accessible through prominent placement and colorful instructional signage. CAT has been found to produce durable functional gains among outpatients with schizophrenia [38].

To date, we are aware of no social cognitive interventions that use physical environmental manipulations to cue adaptive responses. Given the success of CAT in improving domains such as medication adherence and community engagement, environmental supports may be a promising direction for future research on enhancing social cognition. For example, one can imagine a patient having a small card or medallion with the letters “JTC” that she carries in her pocket, and which functions as a frequent reminder to resist jumping to conclusions in social situations (possibly, cuing the thought, “If a person says something unclear, do not assume it is hostile. Ask for clarification.”).

In sum, without the benefit of improved neurocognition, overlearned skills, or environmental supports, it is unclear how in-session gains achieved through social cognitive interventions will be transported to, and maintained within, patients' real-world living environments.

## 6. A Social Psychological Approach to Social Cognition

In an effort to address this issue of treatment effect maintenance, we have recently designed a novel social cognitive intervention that aims to impart durable improvements in ToM, attributional bias, and jumping to conclusions using principles from social psychology. The social psychological literature provides an empirically robust alternative to the *social detective* school of social cognitive training. In fact, fifty years of social psychological research suggests that the *social detective* approach may be incompatible with normal, healthy social cognitive functioning [44]. Further, not only is slow, effortful social cognition abnormal, but the literature suggests that it actually may hinder adaptive interpersonal interaction and reinforce the types of dysfunctional judgments that it was meant to minimize.

The social detective approach has two key problems from a social psychological standpoint. First, it encourages slow, labor-intensive thought. And second, it aims to enable patients to make correct judgments. The problem with encouraging slow, laborious thought is that such thought is experienced by the thinker as difficult, and the social psychological literature clearly shows that when thinking is experienced as difficult, the product of this thought is experienced as bad, invalid, or incorrect. This phenomenon, which falls within the domain of *metacognitive experience* [45], has important implications for clinical intervention.

For example, consider the technique of *generating alternatives * [41], which is widely used in cognitive therapy for psychosis [46] and is a pillar of the social detective approach. *Generating alternatives* is used when a patient is harboring a distorted judgment or belief (e.g., “I know my boss hates me because she passed me without saying Hi.”). By generating alternatives (e.g., “Maybe she was in a rush or did not notice me.”) the patient is led to appreciate that other explanations for an event are possible, diminishing his certainty in his (distorted) interpretation. However, the *metacognitive experience* literature has shown that if the patient experiences the process of thinking up alternatives to be cognitively difficult, this experience in itself will function as evidence against the new alternatives. Experientially, it is as if the patient says to himself, “It was so hard for me to think up alternatives to my initial judgment that my boss *must* hate me—otherwise it would have been easy to think up alternatives!” Thus, ironically, the more alternatives a patient generates, the more convinced he may be of the validity of his initial judgment. Because people with schizophrenia typically have cognitive deficits, they are particularly vulnerable to experiencing the process of carefully, laboriously gathering and evaluating social information as effortful and thus of having *generating alternatives *backfire. Thus, teaching careful social cognition may further entrench social cognitive problems in this population rather than helping them.

A second problem with extant social cognitive interventions is that they teach patients to make “*correct*” judgments. From a social psychological standpoint, this is problematic because normal social cognition is not characterized by accuracy. In fact, average humans' accuracy in judging others' thoughts and feelings hovers around 50% [47]. This poor showing is thought to result from the fact that others' thoughts and feelings are fundamentally intangible to the subject [44]. That is, people are not good at judging others' thoughts because we cannot observe their thoughts. We must make guesses based on limited often misleading, information and thus we are often wrong.

If the social psychological literature suggests that normal social cognition is neither careful nor accurate, then what is it? The evidence suggests that healthy social cognition is characterized by *fluidity*, *flexibility*, and *tolerance of uncertainty* [44]. To operate efficiently in the fast-paced world of social interaction, people foremost are motivated to use a style of social cognition that is quick, efficient, and fluid. Because our capacity for careful, analytic thought is scarce and slow [48], we therefore employ a range of rough-and-ready *heuristics*, or rules of thumb, to enable us to generate serviceable social impressions in real time. In addressing the potential inaccuracy of these impressions, we do not carefully evaluate their empirical support, as social detective training would have us do. Rather, normal people maintain an epistemological stance of openness to uncertainty in which we are willing to abandon one impression and generate a new one in response to changing inputs. Rather than carefully weighing the accuracy of competing impressions, we achieve interpersonal adaptiveness through our ability to flexibly inhabit different perspectives from one minute to the next and to resist committing rigidly to any one.

This social psychological perspective suggests a reframing of the problems of ToM deficit, attributional bias, and jumping to conclusions. ToM deficit may be better understood as reflecting an impoverished ability to generate representations of others' mental states, rather than diminished accuracy [35]. Regarding attributional bias and jumping to conclusions, this social psychological perspective would agree with the social detective approach that rigid adherence to one perspective is problematic, but would differ in its favoring rapid impression formation over slow, careful impression formation.

## 7. A Social Psychological Treatment Approach

Based on this social psychological conception of social cognition, we developed a novel treatment strategy called Mary/Eddie/Bill (MEB). MEB is designed to provide patients with a quick and easy heuristic for generating impressions about others' mental states (to enhance ToM) and for flexibly juxtaposing multiple impressions (to address attributional bias and jumping to conclusions). MEB is based on the *generating alternatives *technique that is used in existing interventions but is modified to make the process more rapid and rote, so that it may be overlearned and to make the experience feel easy rather than difficult, so that its products are judged by the subject to be valid rather than invalid.

To simplify *generating alternatives *we teach patients to generate only three alternatives, and we teach the three alternatives ahead of time in generic form. The three generic alternatives that are taught correspond to three orthogonal attributions that exhaust the universe of potential causes for negative social events. Namely, when a negative or confusing social event happens, one can either blame oneself, blame another person, or blame the situation/bad luck. It is assumed in MEB that patients with ToM deficits have limited capacity to generate these three perspectives, while patients with dysfunctional attributional style and/or a tendency to jump to conclusions tend to rigidly adhere to one of these three prototypical styles at the exclusion of the other two. Thus, some patients rigidly blame themselves across negative situations, conforming to a depressive attributional style [49], some rigidly blame others, conforming to a hostile or externalizing/personalizing attributional style [50] and some rigidly blame situational factors, conforming to responsibility-avoidant style.

To enable patients to easily recall and use these three attributional styles across social situations, each style is taught in the form of a prototypical character who embodies the style's reasoning, emotional, and behavioral characteristics. Thus, *My-fault Mary *always blames herself, feels sad or guilty, and performs actions such as hanging her head, crying, and saying, “This is all my fault!”; *Blaming Bill *always blames other people, feels angry or suspicious, and performs actions such as pointing his finger, glaring, and saying, “You are to blame for this!”; *Easy Eddie *always blames bad luck, tries to feel comfortable and relaxed, and performs actions such as grinning, shrugging, cocking his head, and saying, “Oh well!”

Patients initially practice applying these three characters to pictures and videos of social situations using a forced-choice paradigm (“If you *had to* say, is the person on the left acting most like Mary, Eddie or Bill?”). This simple exercise enables patients with ToM impairment to generate working guesses regarding others' inner states in a way that integrates thought, feeling, and behavior. Because thoughts, feelings, and behaviors form a predictable and coherent scheme within each prototypical character, knowing any of the three domains enables easy identification of the other two (e.g., “He is *acting* like Bill, so he is probably *feeling* angry and *thinking* that somebody has wronged him.”). The forced-choice and exhaustive applicability of the MEB heuristic facilitates generalization across social situations. Patients are taught that in *any* situation, each person can be thought of as most closely matching Mary, Eddie, or Bill. This principle of generalization is practiced in session and homework assignments by assigning the three characters in pictures and videos depicting a wide range of social situations.

To facilitate social cognitive fluidity and flexibility, MEB patients are taught that it is more important to be able to see different perspectives in social situations than to make correct judgments. Thus, after basic MEB training, patients are taught to “flip it,” which consists of imagining how an actor may view a social situation from the perspective of any of the three prototypical characters. For example, “He is smiling, so my first guess is that he is feeling content and not blaming anybody, like Easy Eddie. But if I flip it, he could also be smiling sarcastically, and really be feeling mad at the person on the right and blaming her for causing the spill.”

The aim is for patients to be able to easily recall and use MEB outside of session because of the colorful character prototypes that link action, emotion, thought, and behavior, the characters alliterative names, and the use of overlearning exercises during in-session training.

 We have conducted a 6-session pilot trial of MEB among twenty-four outpatients with schizophrenia or schizoaffective disorder [51]. Posttreatment assessments were conducted one to two weeks after the final MEB session. To assess whether the intervention imparted a memorable cognitive heuristic, patients were asked, “Can you recall the names of the three characters we talked about in the group, and can you tell me how each one of them would think, act and feel?” All 16 treatment completers recalled the three characters' names, 14 were able to accurately recall and assign characteristic feelings and actions, and 13 accurately recalled and assigned all three characteristic attributional styles. To evaluate patients' metacognitive experiences associated with use of the MEB heuristic, completers were asked to rate whether MEB was “hard,” “easy,” or “very easy” to understand and use. None rated it as “hard” and nine rated it as “very easy.” These findings provide initial support for the theoretical model underlying MEB, which aims to teach a heuristic strategy that is memorable enough for patients with schizophrenia and cognitive deficits to recall and simple enough to be experienced as easy.

As a preliminary evaluation of the potential efficacy of MEB, patients also completed measures of social cognition and social functioning at pre- and posttreatment. Although these data must be interpreted very conservatively due to lack of appropriate comparison group and blinding, and small sample size, it is worth noting that statistically significant within-group improvements were observed on measures of ToM, social cognitive overconfidence, and self-reported social engagement [51].

## 8. Conclusion

 Targeted social cognitive intervention for schizophrenia is a young area of inquiry. Extant targeted interventions show promise, but none includes a clear and theoretically supportable model by which maintenance and generalization of gains may be achieved. MEB is a novel targeted intervention that is based on a social psychological model. This model is rooted in robust empirical research and posits a heuristic-based mechanism for generalization and maintenance of gains. Initial research with MEB is promising, but more research is necessary to test this new approach. The need for novel interventions to improve real-world social functioning in schizophrenia is great. To meet this need, treatment developers should build on established intervention techniques, such as overlearning, and also explore novel approaches, such as environmental supports and approaches suggested by allied fields of research, such as social psychology.
